# Application of Machine Learning Models to Identify Differences in Neural Electrophysiological Properties Across Estrous Cycle Phases

**DOI:** 10.1007/s12021-026-09814-0

**Published:** 2026-09-01

**Authors:** Armaan Raina, John Meitzen

**Affiliations:** 1 Department of Biological Sciences, North Carolina State University, Raleigh, NC USA; 2 Center for Human Health and the Environment, North Carolina State University, Raleigh, NC USA; 3 Dept. of Biological Sciences, NC State University, 144 David Clark Labs, Campus Box 7617, Raleigh, NC 27695-7617 USA

**Keywords:** machine learning, estrous cycle, nucleus accumbens, electrophysiology, neuroinformatics

## Abstract

Machine learning models (MLMs) have been used to classify neuron subtypes based upon electrophysiological attributes. What has been less explored is using MLMs to assess differences within a neuron type, especially in the context of subtle changes induced by neuromodulation such as the rodent estrous cycle. Previous research found that the estrous cycle shifts rat nucleus accumbens (NAc) medium spiny neuron (MSN) electrophysiology, including action potential (AP), passive, and miniature excitatory post-synaptic current (mEPSC) properties. This plasticity provides a model system to investigate this question. We hypothesized that MLM classification accuracy would differ when trained across these data types, reflecting the information each encodes regarding estrous phase identity. To test this hypothesis, we extracted electrophysiological features across four estrous cycle phases from a publicly available dataset, and employed this data to train MLMs to classify estrous cycle phase origin. We found: MLMs identified estrous phase origin with up to 94% accuracy (Random Forest); MLM accuracy differed by model and feature set; passive, AP and mEPSC features yielded sequential performances with feature importance analysis revealing input resistance, AP burst length, and mEPSC baseline current as most discriminative for their respective feature sets; the late proestrus phase demonstrated the most distinct electrophysiological profile. These findings demonstrate that MLMs can detect changes associated with the estrous cycle and are useful for assessing electrophysiological differences within a neuron type. They further represent a broader application of MLMs as computational tools useful for understanding the sensitivity of neural properties to the influences of neuromodulatory cycles.

**Supplementary Information:**

The online version contains supplementary material available at https://doi.org/10.1007/s12021-026-09814-0.

## Introduction

Machine learning models (MLMs) have demonstrated great success in classification of neurons across different subtypes (Armañanzas and Ascoli 2015; Barros et al. 2022; Kim et al. 2025; Liu et al. 2025; Ophir et al. 2024; Vasques et al. 2016; Wang et al. 2025a, b), leveraging electrophysiological, histological, molecular, and morphological features to perform this process. However, MLMs have been far less applied to the problem of assessing more subtle, neuromodulatory differences within a neuronal subtype, for example, across natural hormone cycles. Hormone cycles manifest in many forms across vertebrate and invertebrate animals. Examples include testosterone, which exhibits circadian cycles in many animal species, such as male humans and rodents (Hashimoto et al., 2019), while female humans, select rodents, and other species exhibit cyclical alterations in estrogens, progesterone, and other hormones as part of the menstrual and estrous cycles respectively (Itriyeva, 2022; Schwartz, 2000; Somerville, 1971). In rodent models, it is well established that neurons in different brain regions exhibit differential electrophysiological function across estrous cycle phase, including in the hypothalamus, hippocampus, caudate-putamen, and the focus of this study, the nucleus accumbens (NAc)(Chapp et al., 2025; Lewitus et al., 2024; Power & Iremonger, 2021; Proaño et al., 2018, 2020; Quigley et al., 2021; Shanley et al., 2023; C. S. Woolley et al., 1997). In rodents and humans, the NAc is a nexus region between the limbic and premotor systems and is critical for action selection of motivated and reward-driven behaviors, among other tasks, including the motivational components of mating (Floresco, 2015; Tonn Eisinger et al., 2018). The NAc, its associated neural circuit, and component neurons express estrous cycle-relevant hormone receptors (Krentzel et al., 2021; Seib et al., 2023). The most common neuron type in the NAc is the medium spiny neuron (MSN; also called the spiny projection neuron), a GABAergic output neuron which serves as the key computational integrator for this brain region (Klawonn & Malenka, 2019). Thus, any change in the electrophysiological properties of the MSN potentially impacts the outgoing signals from this brain region, and consequently influences behavior and other functions. MSNs have been extensively studied in the context of the estrous cycle, and it is clear that their electrophysiological properties change across cycle phase, namely action potential (AP) properties including the frequency of evoked APs in response to depolarizing current, passive membrane attributes such as input resistance, and miniature excitatory post-synaptic potentials (mEPSC) attributes like amplitude (Proaño et al., 2018, 2020, 2024). This body of data provides a powerful model system for testing whether MLMs are an effective approach for assessing this type of more nuanced change in neuron function.

Existing analysis frameworks have been successful in addressing differences in MSN electrophysiological properties between phases (Long et al., 2023; Proaño et al., 2018, 2020). However, these frameworks are constrained by necessary a priori decisions regarding data analytics approaches, as well as human limitations regarding analyzing the predictive influence of the different types of electrophysiological properties. MLMs provide methods that address these issues (Genkin & Engel, 2020), enabling in-depth understanding of the specific neuronal characteristics that are profoundly impacted by the presence of different hormones across cycle phases. One example of this approach would be an MLM trained to differentiate neuron phenotypes recorded during various estrous cycle phases based on assessments of differential neuron electrophysiological properties, including those of the AP, passive (sometimes called intrinsic or membrane), and synaptic such as mEPSC. This research approach builds upon previous findings where MLMs were employed to differentiate between different types of neurons (Akram et al. 2023; Beau et al. 2025; Wang et al. 2025a, b). Here, classification accuracy could be a useful metric for assessing neuromodulatory influence in the context of the estrous cycle upon neuronal electrophysiological properties, potentially revealing what fundamentally changes regarding neuronal computational function. In other words, determining what aspects of neuronal function are most apparently different to an MLM across estrous cycle phase, which may or may not align with the aspects discerned by human neuroendocrinologists. Furthermore, classification accuracy can be interpreted in the context of the mathematical foundations of the model, where insights into the structure of the neuronal electrophysiological data type can be understood based on which models exceed on which specific feature set.

Thus, we hypothesized that MLMs would be able to differentiate estrous cycle phase origin based upon electrophysiological properties, and that classification accuracy would differ across MLMs as well as feature sets. This approach elucidates the amount of information encoded within each data type with respect to estrous phase, helping understand which particular aspects of cellular function are most modulated. In order to address this research goal, seven MLMs were trained on a feature set extracted from each data type and their accuracies were compared. Furthermore, the feature importances of the random forest classifier (RFC) were explored as a way to uncover which particular characteristics from each feature set were most differentiated across estrous phase. Finally, latent space visualization (LSV) was conducted on each feature set to assess the inherent dimensionality of the data across phase, providing further validation about which data type is most discriminative of estrous phase.

## Materials and Methods

### Dataset Description

The parent dataset motivating this work consists of whole-cell patch clamp recorded MSNs in the acute brain slice preparation of the NAc of adult female rats and was originally published in two manuscripts by (Proaño et al., 2018, 2020) and colleagues. The data are available via the Dryad Data Repository (https://datadryad.org/dataset/doi:https://doi.org/10.5061/dryad.k0p2ngfgm). The data were originally obtained at North Carolina State University and were previously analyzed in published studies employing non-machine learning techniques (Long et al., 2023; Proaño et al., 2018, 2020). All animal protocols were approved by the Institutional Animal Care and Use Committee at North Carolina State University. Briefly, MSN recordings were obtained from adult female Sprague-Dawley rats observed across four assessed phases of the estrous cycle: diestrus, early proestrus, late proestrus, and estrus phase. The parent studies identified estrous cycle phases via analyses of wet mount vaginal cytology and sex steroid hormone plasma concentrations. Only animals that could be clearly assigned to a phase were employed by the parent studies. Diestrus was defined as the ~ 55 h period after estrus phase, when vaginal cytology presents with limited leukocytes, nucleated basophilic cells, and occasional vacuolated cells. Early proestrus phase was defined as near the beginning of the ~ 12-h proestrus phase, typically in the morning of the proestrus phase, when vaginal cytology exhibits only round and nucleated epithelial cells. Late proestrus phase was classified as the time near the end of the ~ 12-h rat proestrus phase, typically in late afternoon in nocturnal animals such as rats, when vaginal cytology exhibits predominantly round and nucleated cells, with initial but minimal appearance of clumped cornified epithelial cells that is characteristic of the estrus phase. Estrus phase is the twenty-five to twenty-seven hour phase that occurs after proestrus. During this phase, vaginal cytology exhibits large basophilic epithelial cells as well as non-nucleated cornified cells, which over time diminish as leukocytes appear. MSN recordings were amplified, lowpass filtered (2 kHz), and digitized (10 kHz) with a MultiClamp 700B amplifier attached to a Digidata 1550 system and a personal computer using pCLAMP 10.7 software. This software generates .abf file types. As previously described (Dorris et al., 2015), recordings were first made in current clamp to assess neuronal electrophysiological properties. MSNs were identified by medium-sized somas, the presence of a slow-ramping subthreshold depolarization in response to low-magnitude positive current injections, a hyperpolarized resting potential more negative than − 65 mV, inward rectification, and prominent spike afterhyperpolarization (Belleau & Warren, 2000; O’Donnell & Grace, 1993).

**Fig. 1:**
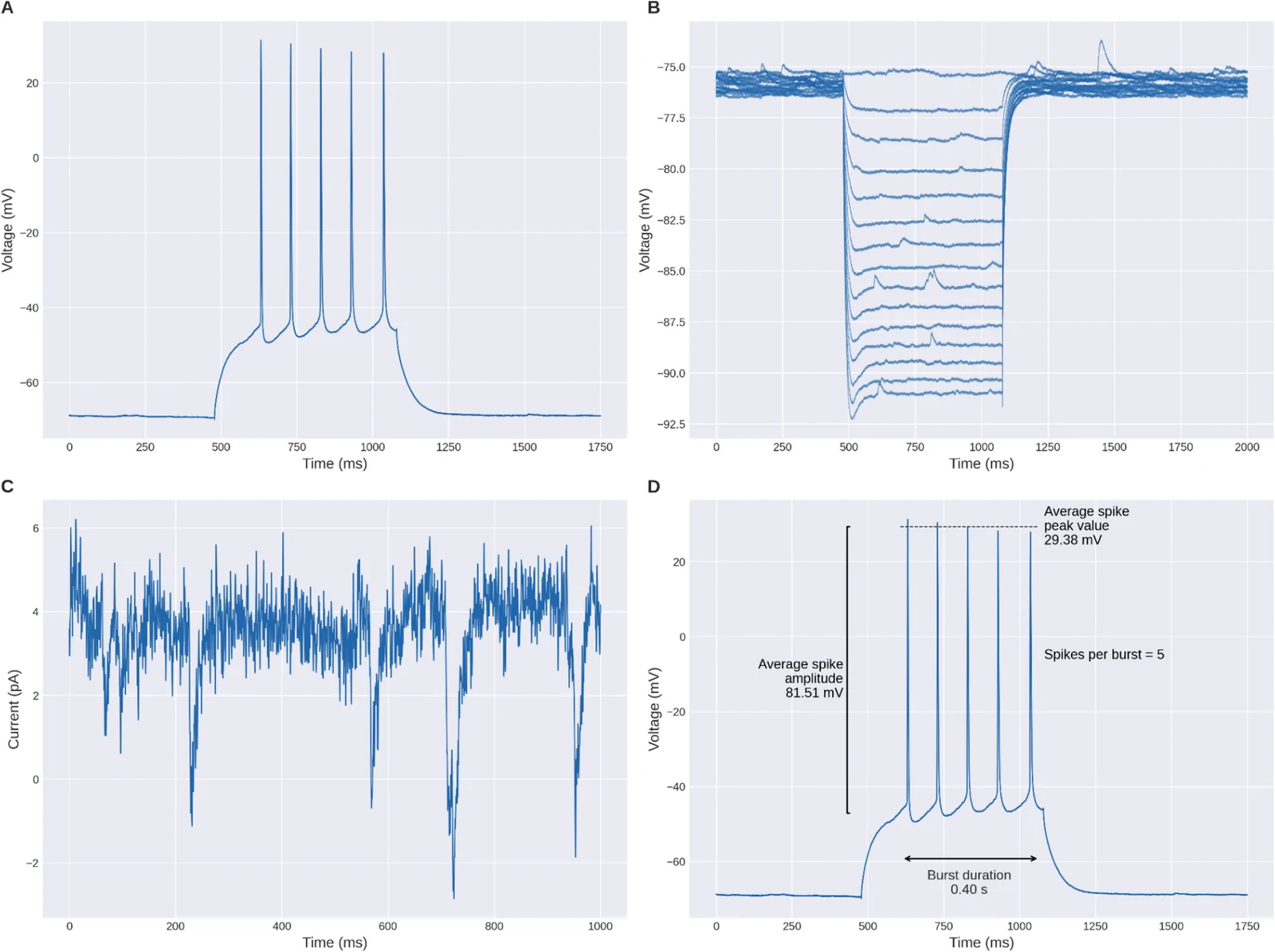
Representative examples of nucleus accumbens (NAc) medium spiny neuron (MSN) electrophysiological recordings. All raw data employed was generated by the parent studies (Proano et al., 2018; 2020) and obtained from the Dryad Data Repository. (**A**) Representative examples of a voltage response of a MSN to a depolarizing current injection, generating information encompassed by the action potential (AP) feature set. (**B**) Representative examples of a voltage response of a MSN to a hyperpolarizing current injection, generating information encompassed by the passive feature set. (**C**) Representative examples of miniature excitatory postsynaptic currents (mEPSCs) recorded in voltage clamp in a MSN with no current injections, generating information encompassed by the mEPSC properties feature set. (**D**) Representative voltage response of a MSN to a depolarizing current injection, annotated with select features relevant to AP burst attribute definitions and criteria used during model training

The dataset is organized into three classes based on the type of MSN electrophysiological property assessed: AP, passive, and mEPSC (Fig. 1). An example of the data collected from a single neuron from a rat in the diestrus phase is provided in Fig. 1. Full details of data collection can be found in the parent studies (Proaño et al., 2018, 2020). Briefly, regarding AP properties (Fig. 1A), neurons were recorded in current clamp configuration, and were injected with excitatory current for 0.6 s and the membrane potential was measured for the duration. The amount of current injected started at 0 ⁢n⁢A, to provide a reading of the baseline resting membrane potential of the neuron since MSNs do not demonstrate spontaneous AP generation, and the depolarizing current was then stepwise increased to observe the complete AP frequency to injected current curve (FI curve). At least three replications were made per neuron, and multiple neurons were recorded across estrous cycle phase (diestrus = 26, early proestrus = 20, late proestrus = 49, estrus = 16). Regarding passive properties (Fig. 1B), neurons were also recorded in current clamp configuration, however were instead injected with inhibitory current for 0.6 s while membrane potential was measured for the duration. The amount of current injected started at 0 ⁢n⁢A, and then the inhibitory current was again stepwise decreased to observe the injected current to delta change in membrane potential (IV curve). At least three replications were made per neuron and multiple neurons were recorded across estrous cycle phases (diestrus = 26 from 11 animals, early proestrus = 20 from 8 animals, late proestrus = 49 from 12 animals, estrus = 16 from 7 animals). Additional details regarding neuron and animal samples are available from the parent studies and associated dataset. Regarding mEPSC properties (Fig. 1C), neurons were recorded in voltage clamp configuration at − 70 mV in the presence of the GABA receptor antagonist picrotoxin and the voltage-gated sodium channel blocker tetrodotoxin to abolish inhibitory postsynaptic current events and action potentials, respectively. mEPSCs were recorded for at least 5 min and multiple neurons were recorded across estrous cycle phase (diestrus = 19, early proestrus = 12, late proestrus = 25, estrus = 14). These settings enable mEPSC recordings from AMPA glutamate receptors exclusively (Nowak et al., 1984; Proaño et al., 2018).

### Computational Environment

All analyses were performed in Python using standard scientific libraries for data processing (SciPy, NumPy, pandas) and model training (scikit-learn). Data loading and feature extraction employed the pyABF and eFEL libraries, respectively. Feature extraction and model training were performed on a high-RAM environment in Google Colab, models were each trained across fixed random seeds for reproducibility.

### Data Preprocessing and Feature Extraction: AP Properties

For AP data, pyABF (Harden, 2022), an open source Python library for reading .abf files, was employed. Relevant .abf files were imported directly into Google Colab where analysis was conducted. Features from 4,073 AP bursts in mV induced by varying amplitudes of injected excitatory current, as described in the dataset description section above, were extracted using the maximum interval method (Cotterill et al., 2016) with a maximum inter-spike interval of 500 ms within a burst selected based on average ISI duration, with code adapted from a publicly available implementation (David, 2023). Figure 1D provides a graphical representation of metrics used to determine burst validity (duration, average spike amplitude, etc.)(Fig. 1D). Briefly, individual APs (also called spikes) were detected using the find_peaks() function within the Scipy library’s signal package. Parameters of this function were specified as follows: the minimum voltage threshold required for a spike was set at -25 mV, the minimum prominence at 15 mV, the minimum spike width at 0.5 ms, and the minimum distance between peaks at 1 ms. Spike widths were measured at half-prominence height. From each detected spike, the following features were extracted: spike peak voltage (mV), spike amplitude relative to the surrounding trough (mV), spike width (ms), rise time to half-prominence (ms), decay time from peak to half-prominence (ms), inter-spike interval (s), and instantaneous firing frequency (Hz). Next, a sample of the diestrus phase data was used to estimate the distribution of the inter-spike interval across a trace, which was found to have a mean of 599 ms and a median of 6.96 ms, reflecting a highly skewed distribution driven by long inter-burst intervals. Based on these findings, the maximum inter-spike interval within a burst was set at 500 ms with a minimum inter-burst interval of 100 ms. Finally, applying these parameters, AP bursts were detected by establishing whether the next spike in a trace belonged to an existing burst or was distant enough in time to be classified as the start of a new burst. The features extracted from this data type used for model training are burst length in milliseconds which is measured as the length of a burst from the start of the first spike to the end of the last, spikes per burst, average spike amplitude in millivolts computed as the prominence (peak event height relative to data points around it) of each spike within a given burst, average spike peaks in millivolts calculated as the average of absolute height of each spike within a given burst, and finally spike frequency.

### Data Preprocessing and Feature Extraction: Passive Properties

Passive membrane files were similarly read into Google Colab using pyabf, where features were extracted from 5,348 traces in mV induced by hyperpolarizing current injection (Proano et al., 2018; 2020) using the eFEL library (Ranjan et al., 2026), adapting the implementation from previously published techniques (David, 2022) alongside a custom implementation of inward rectification ratio, computed by taking the ratio of the resistances between the first and last sweep within a trace. The features extracted from this data type used for model training were obtained from the eFEL library. Briefly, they are defined as follows: the resting membrane potential of the neuron in millivolts computed per trace; the steady-state voltage in millivolts defined as the hyperpolarized voltage at the bottom of negative current injection post sag; the minimum voltage in millivolts achieved in a sweep; voltage deflection onset in millivolts measured as the voltage value at the current step onset; rebound voltage in millivolts represented by any overshoot in voltage above RMP that occurs immediately after the hyperpolarizing current step ends; voltage change computed as the difference in voltage between RMP and steady state; injected current; input resistance which measures the resistance of the membrane to current, computed as steady state voltage divided by injected current; membrane time constant in milliseconds derived by fitting a monoexponential model to the data following the offset of the hyperpolarizing current step; membrane capacitance in picofarads; sag amplitude in millivolts computed as the difference in voltage between the membrane at the minimum voltage and the steady state voltage as induced by hyperpolarizing current injection; sag ratio 2 which is the ratio of voltage sag and negative peak amplitude; and finally inward rectification ratio, computed by dividing membrane resistance values across the first and last current steps. Sag ratio 1, defined as the ratio between the sag amplitude and the difference between RMP and minimum voltage and sweep number were both excluded from the feature set. Sag ratio 1 was specifically excluded due to its perfect correlation with sag ratio 2.

### Data Preprocessing and Feature Extraction: mEPSC Properties

Regarding mEPSC analysis, .abf files corresponding to mEPSC recordings were analyzed using PClamp 10.7 (Molecular Devices) and MiniAnalysis 6.0.7 (Synaptosoft). In PClamp, a lowpass 2000 Hz Gaussian filter was applied to the signal. This filtered .abf file was then imported into MiniAnalysis. In MiniAnalysis, threshold and area threshold were set to 5 pA, and 5 pA*ms respectively following the procedure of the parent study of this dataset (Proaño et al., 2018), allowing for mEPSC event detection and measurements (61,060 mEPSC events). The resulting data was saved into excel spreadsheets. Using the pandas python library, these excel sheets were imported into google colab where non-physiological events were identified (defined as exhibiting an amplitude greater than 50 pA or if the detected preceding baseline current was less than 0 pA) and then excluded from the feature set. The features extracted from this data type and used for model training are as follows: inter-event internal (IEI) in milliseconds; amplitude in picoamps; rise time in milliseconds, the time from the beginning of the mEPSC to the maximum height achieved in milliseconds; decay in milliseconds computed as the time from the maximum height achieved back to the resting value after the event; area (pA*ms) the amount of current underlying an mEPSC event; baseline preceding current before mEPSC event in picoamps; noise in picoamps calculated as the RMS of the signal corresponding to the event; 10–90 rise time in milliseconds, computed as the time in between 10% and 90% of current relative to the max height of the mEPSC; half width in milliseconds computed as the duration of the event above half its peak amplitude; Rise-50 in milliseconds, the time from start of mEPSC event to 50% of its peak amplitude; 10–90 slope (pA/ms), the average slope of the mEPSC event in the 10–90 rise period.

### Model Training and Classification

All features were scaled using Z-score normalization prior to model training using the scikit-learn StandardScaler() function to ensure that model performance was not distorted by the scale or units of the input features. Seven MLMs from the scikit-learn library were trained on each feature set. These were the random forest classifier (RFC), gradient boosting classifier (GBC), logistic regression (LR), k-nearest neighbors classifier (kNN), support vector classifier (SVC), multi-layer perceptron (MLP), and the decision tree classifier (DTC). Each model was trained five times on a list of randomly generated seeds (which remained consistent across the feature sets) to ensure that the default parameters were not generating inaccurate results. We found it acceptable to train these models without hyperparameter tuning because the goal of the study was not to maximize accuracy but rather to use models as tools to gain better insights into the underlying information encoded within the data. In other words, the goal of the study was more to compare feature sets across various models as opposed to optimizing any single architecture. Accuracies displayed throughout this study are the averages of those five trials. The feature set was split 75% for training and 25% for testing. Each model was trained to classify estrous phase (diestrus, estrus, early proestrus, and late proestrus) given a feature set, thus a total of 21 models were trained. Methods for label randomization controls were likewise derived from previously established techniques (Ojala & Garriga, 2009).

### Latent Space Visualization Analysis

In order to obtain a graphical understanding of the data, a technique to visualize each feature set in a lower dimensional space called t-SNE was utilized (Maaten & Hinton, 2008). t-SNE enables a lower dimensional visualization of each feature set by transforming the data from its natural dimension into a lower, more interpretable dimension by creating a distribution representing the distance of each point from all of the others and then projecting that into a lower dimension. Using the TSNE() function from the scikit-learn.manifold package, each feature set was visualized in three dimensions. The resulting visualizations were generated using a perplexity of 25 given 300 iterations to converge.

### Feature Importance Analysis

The RFC computes a measure of class impurity with respect to each feature as a part of its classification process (Breiman, 2001), generating which specific features had values that clearly partitioned the different phases of the estrous cycle. Briefly, this analysis determines the importance of a feature by calculating the mean decrease in impurity with respect to the target variable. This analysis is provided by accessing the scikit-learn RandomForestClassifier.feature_importances_ attribute. For each feature set, the RFC was either the highest performing or second highest performing model, thus, the assessed importances are reflective of a classification process that accurately captured the differences across phases.

### Statistics

Data were analyzed as appropriate with either one-way or two-way ANOVAs with Newman-Keuls and Tukey’s HSD post-hoc tests respectively. All statistical tests and analyses were conducted using the statsmodel package in Python. P values < 0.05 were considered a priori as significant. Data are presented as means ± standard deviation.

## Results

### MLMs can Successfully Identify Estrous Cycle Phase via MSN Electrophysiological Features, Albeit with Differential Accuracy Depending upon Feature Set and Model Architecture

**Fig. 2:**
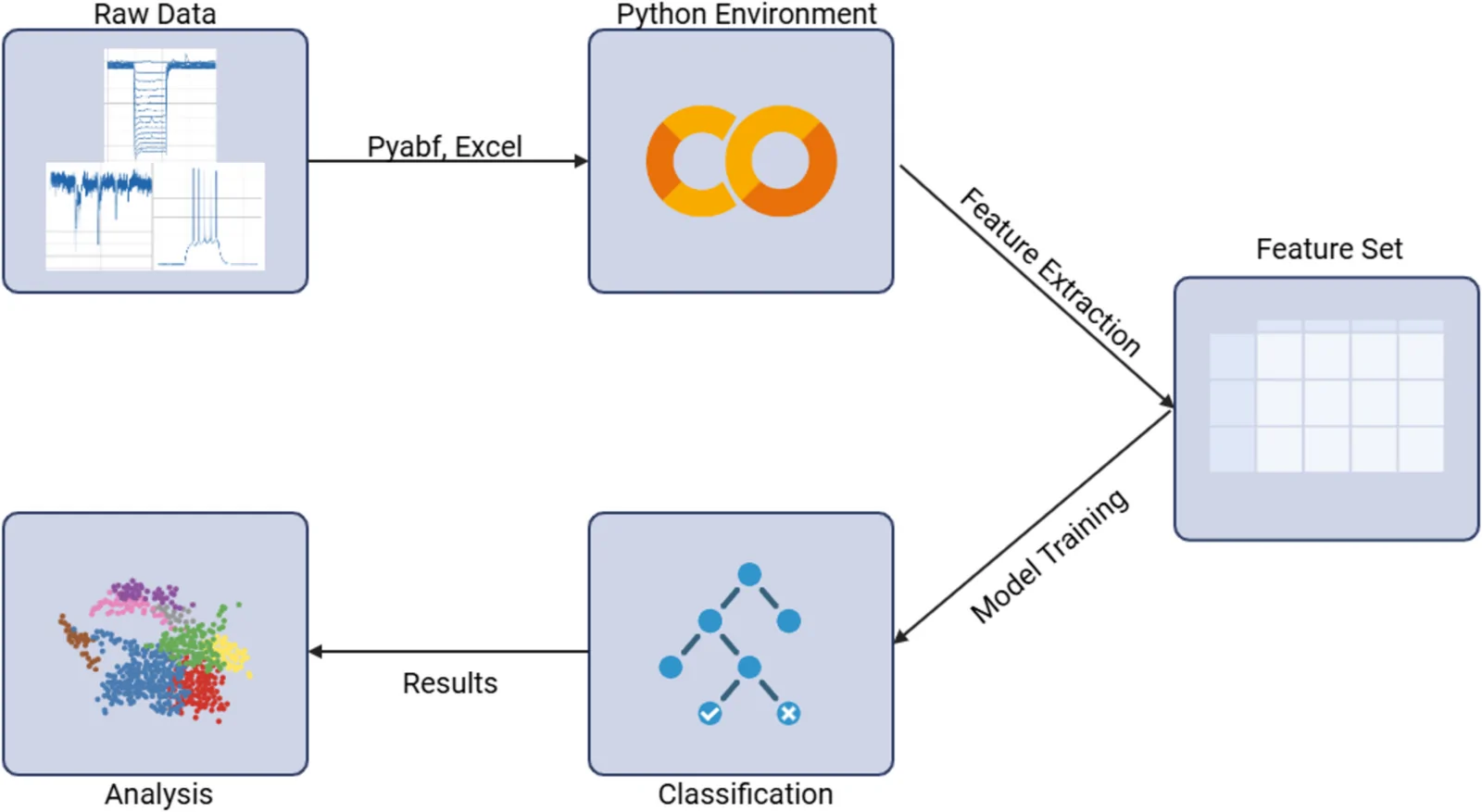
Computational pipeline employed by this study. Feature extraction from the AP, passive, and mEPSC properties were obtained from raw data and analyzed in a Python environment. Seven MLMs were trained using each feature set and results analyzed using relevant techniques. Image made using BioRender

**Fig. 3:**
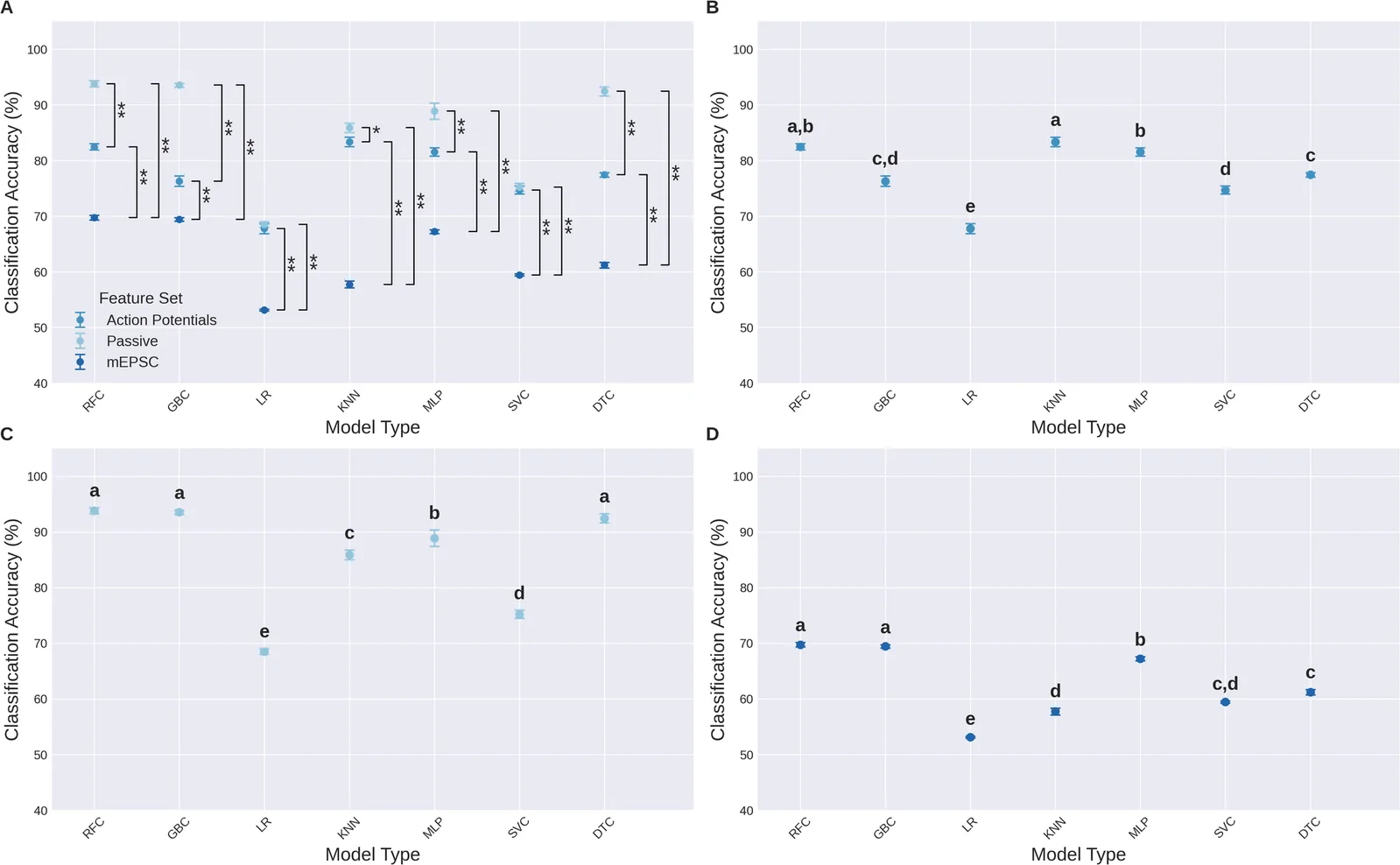
MLMs successfully identify estrous cycle phase when trained with MSN electrophysiological features, albeit with differential accuracy depending upon feature set type and model architecture. MLM accuracy differed significantly by model type, feature set, and interaction (Model Type: F_(6, 84)_ = 1121.962, *P* < 0.0001; Feature Set: F_(2, 84)_ = 8146.1, *P* < 0.0001; Interaction: F_(12, 84)_ = 162.0, *P* < 0.0001). For panels B, C, and D, different letters denote significant differences between groups. (**A**) MLMs trained on passive properties generally exhibited the greatest classification accuracy, followed by those trained with action potential (AP), and mEPSC properties. Within each model, post-hoc testing revealed that the mEPSC feature set yielded the lowest classification accuracy, differing significantly from the higher classification accuracies generated by the passive and AP feature sets (*P* < 0.001 for all). (**B**) MLM accuracy trained on AP feature set. Classification accuracies varied by MLM. (**C**) MLM accuracy trained on passive feature set. (**D**) MLM accuracy trained on mEPSC feature set. Acryonyms: * = *P* < 0.001; ** = *P* < 0.0001;

To test the hypothesis that MLMs are capable of classifying estrous cycle phase based on features extracted from different electrophysiological recording types, we employed seven different MLMs, each of which were trained to classify estrous phase (Fig. 2). Each model was trained five times over random seeds using one of three different feature sets: AP, passive, and mEPSC (Fig. 3A). MLM accuracy differed substantially by model type, feature set, and interaction (Model Type: F_(6, 84)_ = 1121.962, *P* < 0.0001; Feature Set: F_(2, 84)_ = 8146.1, *P* < 0.0001; Interaction: F_(12, 84)_ = 162.0, *P* < 0.0001). Within each model, post-hoc testing revealed that the mEPSC feature set yielded the lowest classification accuracy, differing significantly from the higher classification accuracies generated by the passive and AP feature sets (*P* < 0.0001 for all). Classification accuracies generated by the AP feature sets (Fig. 3B), passive feature sets (Fig. 3C) and mEPSC feature sets (Fig. 3D) also all varied by MLM. Regarding passive features in particular, the RFC exhibited highest classification accuracy at 93.81 ± 0.54%, followed by the GBC at 93.55 ± 0.36%. The lowest accuracy MLM trained on the passive features was the LR at 68.53 ± 0.50%. For the AP features, the kNN performed best, classifying estrous phase with 83.36 ± 0.87% accuracy, followed by the RFC at 82.47 ± 0.57%. The worst performing model was the LR which achieved 67.77 ± 0.91% classification accuracy. Regarding mEPSC features, the top two models were the RFC and the GBC with 69.75 ± 0.40% and 69.44 ± 0.31% classification accuracies respectively, while the worst performing model was the LR at 53.15 ± 0.17%. To verify that models were accurately assessing the patterns underlying estrous cycle origin, models were retrained with scrambled labels and their accuracies were analyzed. If MLMs were assessing true patterns within each respective feature set, then accuracy would be expected to decrease when presented with randomized data. Classification accuracies were significantly lower on the label-randomized training data compared to the normal training data (F_(1, 168)_= 21528.4, p < 0.0001), confirming that the MLMs learned real patterns. These data indicate that the best performing MLMs can classify estrous cycle phase with high accuracy, and that MLM accuracy differed substantially by model type and feature set. Across the respective highest performing MLMs, passive features yielded highest performance, followed by AP features, and mEPSC features.

### Feature Importance Analysis Identifies AP Burst Length, Input Resistance, and mEPSC Baseline Current Level as Most Informative for Estrous Cycle Phase Classification

**Fig. 4:**
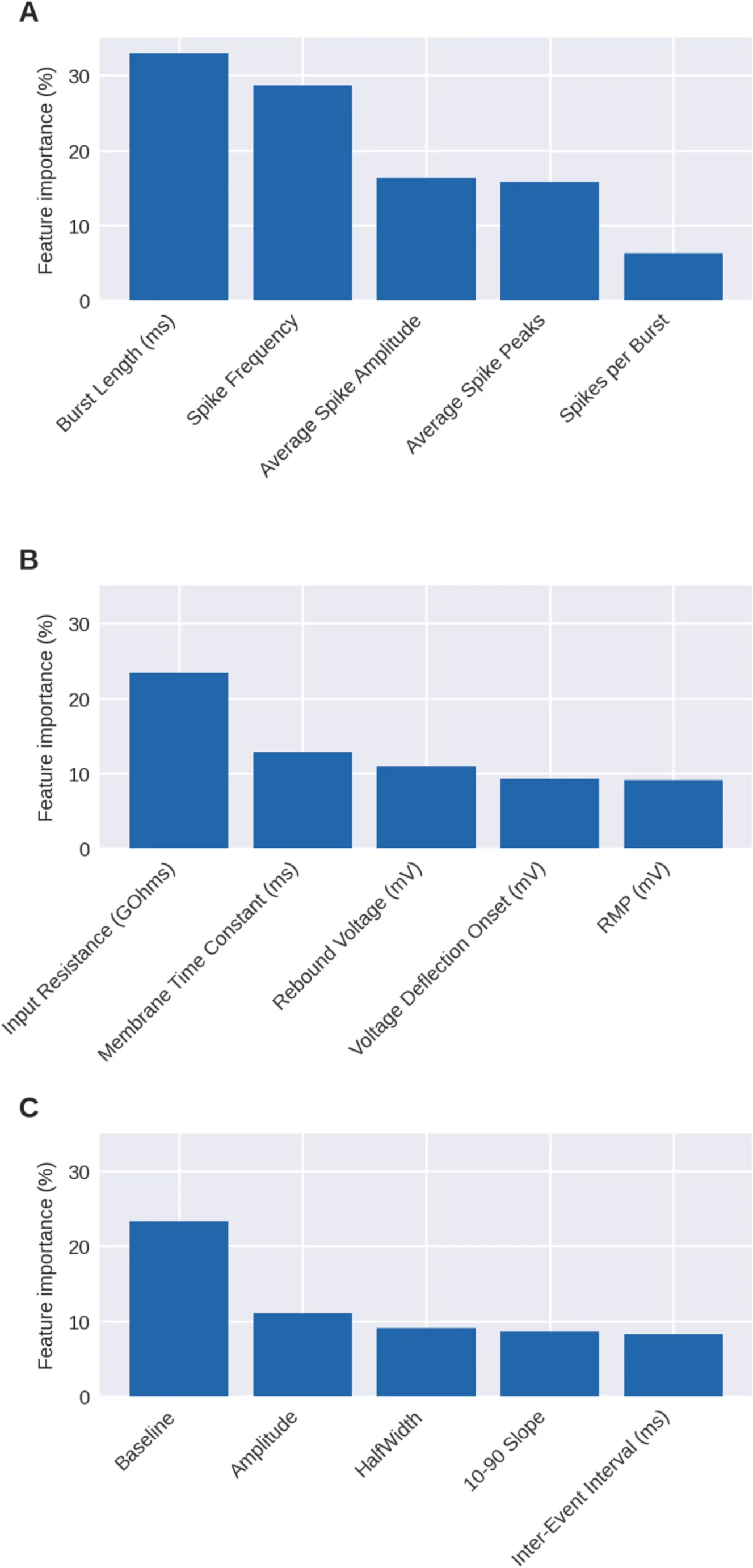
Feature Importance Analysis (FIA): comparison of the top five most important features detected by the Random Forest Classifier trained on AP, passive, and mEPSC feature sets. (**A**) AP features. AP burst length exhibited the highest feature importance. (**B**) Passive features. Input resistance exhibited the highest feature importance. (**C**) mEPSC features. mEPSC baseline current exhibited the highest feature importance. Please see Table 1 for complete FIA data as well as the Results Section. Acronyms: Avg., Average; RMP, resting membrane potential

In order to further explore the relationship between specific features and model output, we then assessed feature importances of the RFC. Unlike other MLMs, as part of the classification process, the RFC computes the gini index of each feature, a measure of impurity (entropy) that quantifies how much a given feature reduces uncertainty in class assignment across the variable of interest, estrous cycle phase in this case. This tool allows identifying the contribution of each feature to classification decisions, showing which characteristics of a given data type were modulated most noticeably by the change in estrous cycle phase. This information is of utility as it helps explain which of the provided features the RFC identified as most discriminative in determining the estrous cycle phase of a neuron. For each data type, every available feature was assessed (AP = 5, passive = 13, mEPSC = 12). All resulting data are provided in table form (Table 1), and the top five features are graphed (Fig. 4). We note that select AP features used the term “spike” synonymously with AP. For the AP feature set, burst length in milliseconds was the most discriminative across estrous phases, closely followed by spike frequency. The lowest assessed importance for the AP feature set was the number of spikes per burst. For the passive feature set, input resistance was by far the most important feature, followed by membrane time constant, with the least important feature being steady state voltage. Finally, for the mEPSC feature set, baseline current level was the most important feature, followed by amplitude; the least important feature was noise.

**Table 1:** Feature importances for each computed feature set expressed as a percentage of mean decrease in label impurity

Action potential	Importance	Passive	Importance	mEPSC	Importance
AP Burst Length (ms)	32.91%	Input Resistance (GΩ)	23.40%	Baseline	23.31%
Spike Frequency	28.67%	Membrane Time Constant (ms)	12.79%	Amplitude	11.11%
Avg Spike Amplitude (mV)	16.38%	Rebound Voltage (mV)	10.91%	HalfWidth	9.11%
Avg Spike Peak (mV)	15.77%	Voltage Deflection Onset (mV)	9.31%	10–90 Slope	8.60%
Spikes per Burst	6.27%	RMP (mV)	9.11%	Inter-Event Interval (ms)	8.29%
		Inward Rectification Ratio	7.11%	Area	8.18%
		Capacitance (pF)	6.67%	Decay (ms)	7.47%
		Current (pA)	5.26%	10–90 Rise	6.72%
		Sag Amplitude (mV)	4.83%	Rise50	6.43%
		Sag Ratio 2	2.82%	Rise (ms)	5.70%
		Steady State Voltage (mV)	2.61%	Noise	5.07%
		Min. Voltage (mV)	2.60%		
		Voltage Change (mV)	2.57%		

### Latent Space Visualization Reveals Stronger Differentiation Across Estrous Cycle Phase for AP and Passive Properties Compared to mEPSC Properties

**Fig. 5:**
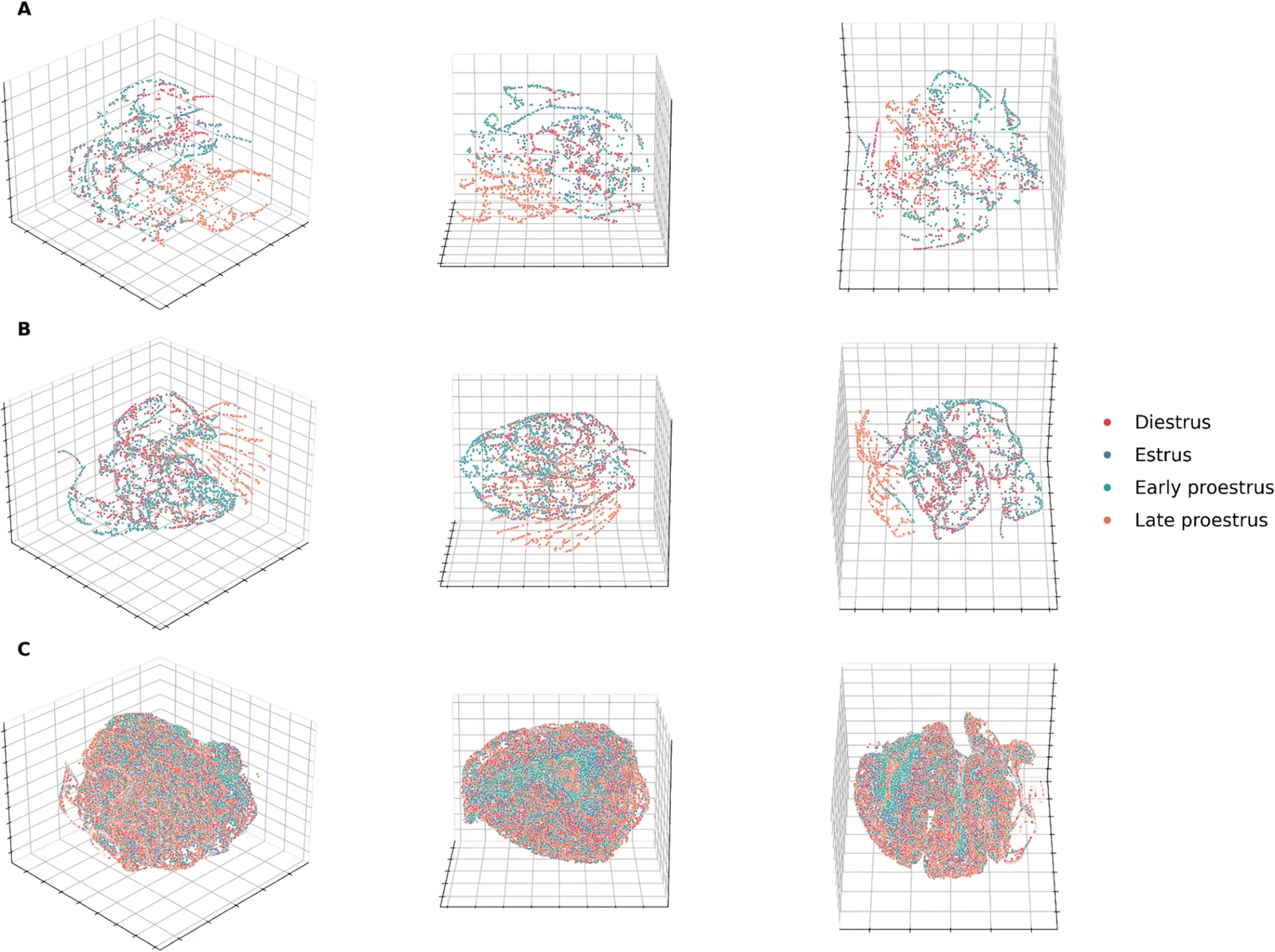
Three-dimensional t-SNE visualization of each feature set color coded by estrous cycle phase. Each visualization is rotated in space for perspective from the left, center, and right perspective. Axis labels are arbitrary, representing t-SNE components 1, 2, and 3 respectively. (**A**) AP features. Early and late proestrus phases are clearly separated compared to other phases. (**B**) Passive features. Early and late proestrus phases are clearly separated compared to other phases. (**C**) mEPSC features. This feature set displays the least separation between estrous cycle phases

The analyses above indicated that passive and AP properties yielded substantially higher classification accuracy than mEPSC features. This finding suggested that a visualization analysis may qualitatively detect greater demarcations in AP and passive properties assessed between estrous cycle phases compared to mEPSC properties. To achieve this goal, t-distribution stochastic neighbor embedding (t-SNE) was employed to reduce the high dimensional data associated with this analysis into a lower dimensional space. Each feature set (AP, passive, and mEPSC) was reduced to three dimensions and visualized across estrous cycle phases. When visualizing the AP properties (Fig.5A), it is clear that the feature set effectively differentiates between late proestrus and the remaining three phases, indicating that AP properties are most distinct during late proestrus, with the remaining three phases showing greater overlap in low dimensional space. Likewise, for the passive feature set (Fig. 5B), there is clear distinction between the late proestrus phase and the other three phases, again emphasizing the uniqueness of the late proestrus phase. Finally, for the mEPSC feature set (Fig. 5C), there appear to be diffuse striations in the visualization, however ultimately distinction is lacking compared to the other feature sets, indicating that mEPSC properties do not vary across phases in a way that produces as meaningful a separation in lower-dimensional space. Overall, visualization in low dimensional space reveals that both the AP and passive feature sets exhibit qualitative demarcations between estrous cycle phases, consistent with the results from the employed MLM techniques. Thus, latent space visualization serves as a key confirmation that application of MLMs and other techniques can differentiate between MSN electrophysiological properties recorded from different estrous cycle phases.

### Per-Class Recall Analysis Indicates that the Late Proestrus Phase was More Readily Distinguishable Compared to Other Phases

**Fig. 6:**
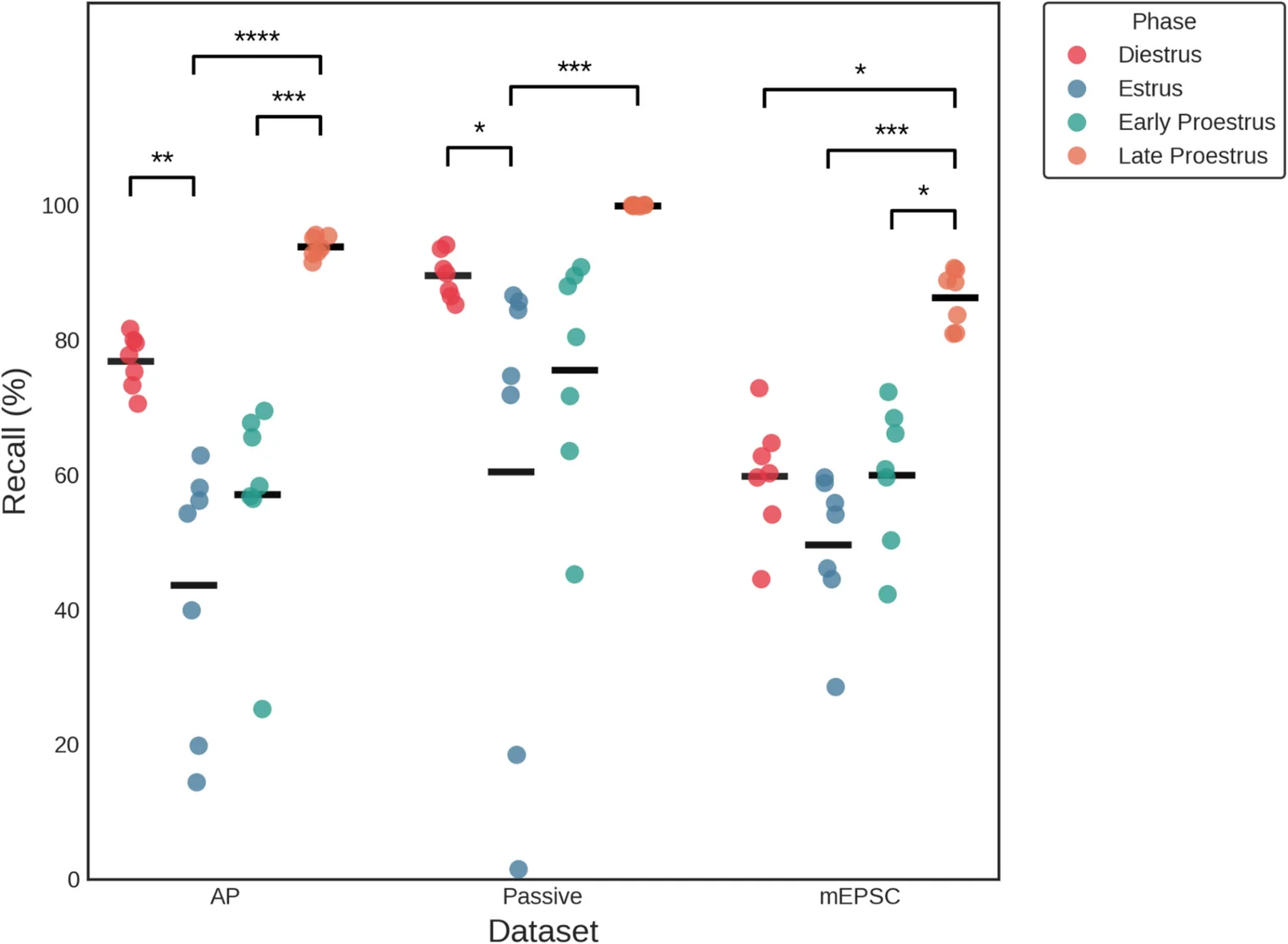
MLM per-class recall across the AP, passive, and mEPSC feature sets. Across all data feature sets and MLMs, late proestrus phase (orange) consistently exhibits the highest recall, while estrus phase (blue) lags below the other phases. Diestrus phase (red) and early proestrus phase (green) values are intermediate and display considerable variation across MLMs. Across all MLMs and feature sets, the late proestrus phase significantly differs from the other phases (Model Type: F_(6, 36)_ = 12.23, *P* < 0.0001; Feature Set: F_(2, 36)_ = 31.70, *P* < 0.0001; Phase: F_(3, 36)_ = 90.35, *P* < 0.0001). Please see the supplemental materials for full post-hoc analysis. Black horizontal bar indicates mean. * = *P* < 0.05; ** = *P* < 0.01; *** = *P* < 0.001; **** = *P* < 0.0001

Given the interest of this study in determining the discriminative capacity of the various feature sets as well as how well MLMs were able to predict the real instances of each class, the per-class recall (a metric that specifically computes how many of the instances of a class present in the data were correctly predicted) was analyzed (Fig. 6). Across all MLMs and feature sets, the late proestrus phase is clearly distinguished from the other phases (Model Type: F(6, 36) = 12.23, P < 0.0001; Feature Set: F(2, 36) = 31.70, P < 0.0001; Phase: F(3, 36) = 90.35, P < 0.0001). The pairwise multiple comparisons are included in supplemental materials (Table S1). This is evident from the recall values observed: 93.84 ± 1.44% when trained on AP data, 99.92 ± 0.09% on passive data, and 86.28 ± 3.99% on mEPSC data. The estrus phase exhibited the worst recall across models: 43.61 ± 18.07% when trained on AP data, 60.43 ± 32.66% on passive data with the LR trained on this data correctly assessing estrus recordings only 1% of the time, and 49.61 ± 10.18% on mEPSC data. As for the other two phases (diestrus and early proestrus), their values ranged between these two. Diestrus phase models achieved a recall of 76.84 ± 3.71% when trained on AP data, 89.57 ± 3.17% on passive features, and 59.80 ± 8.19% on mEPSC features. Early proestrus phase models exhibited a recall of 57.05 ± 13.91% when trained on AP data, 75.57 ± 15.52% on passive data, and 59.95 ± 9.77% on mEPSC data. These results demonstrate that MLMs across all data types more accurately classified late proestrus data points than those recorded from any other phase by far, with recordings from estrus phase presenting the worst recall and correspondingly least electrophysiological consistency.

## Discussion

This study makes three principal findings. First, select MLMs can be trained to successfully classify the estrous phase origin of a neuron with accuracy varying by feature set and model type. The RFC featured the highest performance, particularly when trained on passive properties. Second, feature importance analysis revealed that AP burst length, input resistance, and mEPSC baseline current were the most salient features for their respective feature sets. Third, a review of model classification reports provided insight into the consistency and distinctiveness of the electrophysiological signature of each estrous cycle phase, demonstrating that the late proestrus phase exhibits the most identifiable electrophysiological signature. To our knowledge this is the first demonstration that MLMs can differentiate between neuron electrophysiological properties recorded between estrous cycle phases, not only in the NAc but also for any other brain region. More broadly, this finding indicates that machine learning techniques can be applied to differentiate subtle changes in neuron electrophysiological biophysics induced by neuromodulator action.

### Passive Properties

All 7 models demonstrate the same classification accuracy hierarchies: passive, AP, and then mEPSC. One interpretation of this finding, which we favor, is that at least from the point of view of the MLMs that passive features ultimately encoded the most information about estrous cycle phase identity. Interestingly, while passive features generated the highest accuracy values across all employed MLMs, they also generated the largest range of accuracies across models. This indicates that the data points may exhibit complex, non-linear separation boundaries in the passive feature space, an interpretation visualized in a lower dimension space (Fig. 5B). This conclusion is further validated by the behavior of the LR and SVC. The LR and SVC models produce lower accuracies when trained on passive properties. Both models compute a linear separation boundary between classes (Cortes & Vapnik, 1995), a computational approach that would struggle with the non-linear nature of this feature set. Consistent with this conclusion, the strength of ensemble models including the RFC and GBC naturally suggests a non-linear pattern within each feature set, most applicable to the passive feature training set.

A major portion of the training process for a RFC includes the computation of a metric that captures how well a given feature separates the variable being classified (Breiman, 2001). From a biological standpoint, a property such as input resistance that better distinguishes estrous cycle phase origin means that the property features more distinct values across estrous cycle phases. In turn, this indicates that the feature property, again most prominently input resistance, is likely more sensitive to the relevant hormone action, a prediction which was born out by estradiol replacement experiments in ovariectomized animals as discussed further below (Proaño & Meitzen, 2020). Consistent with this conclusion, the high information content of the passive feature set is consistent with the analysis performed by the parent studies, which found greatest changes across estrous cycle phases in the passive and AP properties (Proaño et al., 2018, 2020), including input resistance in the linear and rectified ranges. Regarding the current study, in the passive feature set, feature importance analysis found that input resistance was the most important feature by far, almost doubling in value compared to the penultimate property, membrane time constant. Differences in input resistance across estrous cycle phases have been well documented in NAc MSNs. NAc MSNs recorded in early proestrus, late proestrus, and estrus phases exhibited cyclical changes in input resistance and other passive and AP properties across phases (Proaño & Meitzen, 2020; Proaño et al., 2018; Oginsky & Ferrario, 2019). In ovariectomized female rats, exogenous systemic estradiol replacement calibrated to carefully mimic the temporal and circulating estradiol concentrations modulated NAc MSN input resistance in both the linear and rectified ranges (Proaño et al., 2020), but did not impact other properties captured by the mEPSC recordings which were less effective in training the MLMs. These less influential features such as mEPSC-derived ones are regulated by fast changes in acute estrogen action, suggesting a more dynamic regulation (Krentzel et al., 2019; Miller et al., 2023). Overall, we conclude that passive properties generated the most powerful data available for MLM-mediated sorting of NAc MSNs across estrous cycle phases.

### AP Properties

Across all MLMs, training with AP properties consistently generated ~ 70% to ~ 85% accuracy. This consistency from model to model indicates that training with AP properties produced a more consistently generalizable representation of the differences across phases, with perhaps some struggle to differentiate certain estrous cycle phase subsets of the data. The power of the AP feature set is consistent with the parent studies, which found that estrous cycle phase as well as circulating levels of plasma estradiol and progesterone regulate AP threshold, and other relevant properties like resting membrane potential and input resistance (Proaño et al., 2018; Proaño & Meitzen, 2020). These properties could be directly induced by hormones, either acutely (Mermelstein et al., 1996) or through changes in ion channel gene expression or membrane placement, or indirect actions through a different neuromodulator, such as dopamine which displays the cycle phase variation (Becker & Cha, 1989; Calipari et al., 2017). In the AP feature set, feature importance analysis indicated that AP burst length, frequency, and average amplitude were the three most important features in MLM training. That these three features account for over 75% of the model’s explanatory power suggests the estrous cycle’s influence on MSN AP properties is broader than each one of these specific features alone, and most likely influences a combination of them that is likely differential across estrous cycle phase. In the parent study multiple AP properties were detected as changing across estrous cycle phases (Proaño et al., 2018, 2020), with some phases showing more distinct influences on AP properties than other phases. This interpretation is consistent with the analysis generated using the classification_report() function in the scikit-learn.metrics package. The models trained on the AP features performed much better on the late proestrus and diestrus phases than either the early proestrus or estrus phases. This is not surprising as these estrous cycle phases generated the most distinctively different MSN properties as detected by the parent studies (Proaño et al., 2018, 2020). We thus conclude that AP properties remain an influential feature set for training MLM to distinguish across estrous cycle phases, and may demonstrate a different dynamic pattern compared to passive properties; a finding not detected by the parent studies, which largely concluded that AP and passive properties demonstrated concomitant dynamics.

### mEPSC Properties

MLMs trained with mEPSC properties yielded the lowest classification accuracies. This result was surprising to us, given that the parent studies found that mEPSC properties, including frequency and amplitude, differed across estrous cycle phases, particularly in the late proestrus phase, and that mEPSC properties are rapidly sensitive to acute, topical estradiol exposure in the NAc (Krentzel et al., 2019; Proaño et al., 2018, 2020). Even the best MLMs trained with mEPSC properties, the GBC and RFC, generated only ~ 70% classification accuracies. The worst performing model in this entire study was the SVC trained on mEPSC properties, demonstrating a 53.15% classification accuracy. MLMs trained using mEPSC properties largely did not exhibit substantial distinction between different estrous cycle phases in terms of model recall. Even more interestingly, for the mEPSC feature set, feature importance analysis indicated that baseline current level assessed *before* the mEPSC event was the most informative feature accounting for over double the separation power as the next most important feature, mEPSC amplitude (pA). This difference in baseline may be indicative of the differences in resting membrane potential across estrous cycle phases (Proaño et al., 2018, 2020). MSNs with varying resting membrane potentials would exhibit different output currents when voltage clamped at -70 mV as part of the protocol used to assess AMPA-mediated mEPSC events in the parent studies. Following up on potential differences in the ion channels potentially underlying this effect is one avenue of future research. MSNs also receive tonic inhibitory GABAergic input (Ade et al., 2008), however this is likely not an influential factor given that the mEPSC recordings were performed in the presence of a GABA receptor inhibitor, and that − 70 mV is near the equilibrium potential for chloride.

One interpretation of the findings of this study may be that AP and passive properties exhibit larger magnitude and more dynamic changes than those manifested by mEPSC properties, or at least changes that are more easily detected by MLM analysis. Consistent with this interpretation are differences in the magnitude and variability of mEPSC attributes compared to those associated with AP and passive properties as calculated from the data presented in the parent study (Proaño et al., 2018). For instance, input resistance in the linear range, the most valuable factor identified by the feature importance analysis, demonstrated a ~ 32% difference in mean value and similar variability between the diestrus and early proestrus phase. In this case, the findings of the parent study align well with the findings of this study. Regarding AP properties, AP width, a factor contributing to the AP Burst Length and Spike frequency analyses identified as important by the current study, demonstrated a ~ 9% difference in mean value concomitant with decreased variability between the diestrus and early proestrus phase. Here a clear advantage of the MLM approach is evident, as the analysis was able to detect a larger impact of AP properties than the parent study. Regarding mEPSC amplitude, the second most important factor identified by feature importance analysis, this attribute demonstrated a similar ~ 9% difference in mean value but exhibited a much higher variability than input resistance or AP width. This increased variability likely impacted the MLM analysis conducted here. Indeed, the MLMs trained on mEPSC data more easily distinguished the late proestrus phase from any other, similar to those trained on the AP and passive feature sets, perhaps indicating more specific phase impacts, a more nuanced interpretation which we favor as being more likely. Specifically, the largest estrous-cycle related impact on mEPSC properties is on mEPSC frequency in the late proestrus phase. This may be too granular a difference for the models to detect without more training data. An additional consideration is within-phase variability, which could also play a role in the success of MLM performance. The parent study did not distinguish day of diestrus or estrus phase, which could be relevant as these phases extend for more hours than the proestrus phases. Further exploration of synaptic properties manifested either as mEPSC or other data types should be an active area of future research. One interesting avenue would be to further examine the temporal aspects of the mEPSC data, as the current study employed a different computational approach to assess frequency than the parent studies. This study analyzed mEPSC interevent interval rather than calculating a grand mean of the overall frequency. We thus recommend that further studies continue to assess the interactions between MLMs and excitatory synaptic activity.

### Classification Reports Across all MLMs and Data Types Reveal a Consistent Electrophysiological Signature of Each Estrous Cycle Phase

The per-class recall metric indicated that across all models and all feature set types (including mEPSC properties), the late proestrus phase is most distinguished from the rest. This is evident from the data presented in Fig. 6, showing recalls which ranged from 93.84 ± 1.44% trained on AP data, 99.92 ± 0.09% on passive data, and 86.28 ± 3.99% on mEPSC data. This consistent high performance is indicative of an extremely distinct electrophysiological signature between phases. From a biological perspective this is consistent with the highly dynamic endocrine action occurring during this restricted phase, with acute impacts from estrogens, progesterone and other hormones that act on the NAc and other brain regions to increase exploratory behavior, decrease anxiety-like behavior, and increase the motivation to mate. Interestingly, the next cycle phase, the estrus phase, exhibited the least distinct electrophysiological signature as the recall across MLMs were 43.61 ± 18.07% when trained on AP data, 60.43 ± 32.66% on passive data, and 49.61 ± 10.18% on mEPSC data. This lack of success may be indicative of the transitional nature of estrus phase in the context of NAc physiology. For the NAc, estrus phase is a period of time when some hormone effects on MSN physiological properties are still present while others are gone or changing back to diestrus phase phenotype, as the NAc is only one component of the lordosis circuit which is primarily situated in the hypothalamus. The other two phases, diestrus phase and early proestrus phase, generate more accurate per-class recall metrics that fall intermediate between the late proestrus phase and the estrus phase. Diestrus phase values are only moderately lower than late proestrus, representing the stability of NAc MSN electrophysiological properties during this multi-day phase when the animal does not display interest in mating. The early proestrus phase exhibits the early impact of endocrine action, before full manifestation, so it makes sense that the associated per-class recall values would be intermediate. These specific findings, driven by the per-class recall metrics, thus provide potential insight into the underlying biology of this system and could perhaps be usefully expanded to analyze the physiology of other relevant brain regions.

### Limitations and Future Directions

One important limitation to this study is that the parent dataset exhibits considerable class imbalance: far more data was available for the late proestrus stage compared to other estrous stages. Consequently, more data points were computed for the late proestrus phase and models may have been prone to overfitting to this phase. This numerical variability is common in manual whole-cell patch clamp studies, given their low throughput/high resolution nature (Hamill et al., 1981), especially in the context of studies of naturally cycling preclinical animal models. Nevertheless, in order to help address this concern, all models were retrained on a subset of the feature set containing an even number of data points per phase. An ANOVA with Tukey’s HSD post hoc test was conducted to determine if the accuracies between the models trained on the full training dataset compared to the numerically constrained training dataset were significantly different. The results found no significant difference in accuracy between the full and even label training datasets within the same data type across all MLMs (analysis not shown). This lack of finding indicates that the class imbalance caused by an increased amount of late proestrus data did not systemically bias the performance of the MLMs. Another relevant limitation is that MLM training depended upon the a priori selection of electrophysiological properties of interest. While all selected training properties have strong neuroscientific justification, this approach does inherently limit training to these select, assessed properties. While a previous analysis of the NAc MSN AP dataset that employed a statistical approach that did not employ a *priori* defined metrics found similar results to both the parent studies and the current study (Long et al., 2023), it is possible that a MLM could find more value from metrics not assessed. The analysis by Long and colleagues additionally analyzed the hierarchical nature of the dataset, including the role of the number of neurons recorded from the same animal, and still found the same conclusions as the parent studies. Overall, an important future direction may be combining analysis techniques that do not pre-select metrics of interests along with MLM analysis, as well as incorporating additional variables.

We also note that this study was specifically designed to assess differences between the different types of neuronal electrophysiological properties assessed by the parent studies: AP, passive and mEPSC. This approach has allowed us to elucidate the informational capacity of each of these data types, however, it does not permit combinatorial comparisons. Thus, questions of whether combining these datasets would enable higher training values are not addressed. Given that the current study achieved high identification rates with select electrophysiological steps, the question of whether combining these different types of electrophysiological feature sets would further enhance accuracy may be of less value to pursue. However, given that there are also select anatomical changes in MSNs across estrous cycle, sex, region and other relevant variables (Beeson & Meitzen, 2023; Dale et al., 2025; Meitzen et al., 2011), it may be of more interest to combine morphological and electrophysiology metrics, as well as transcriptomic data as it becomes more available for this region and estrous cycle phase. These types of data have all been used in MLM-driven neuron classification analyses (Akram et al., 2023, 2023; Mao & Staiger, 2024; Spalińska et al., 2026; Vasques et al., 2016; Vasques & Cif, 2025), both alone and in combinatorial data analytic pipelines. Thus, this type of methodology could further be applied to entirely different types of data (genetic, histological, imaging) or across different types of cycles (circadian, seasonal, etc.) to uncover the influence of various experimental conditions on single neuron or network function, similarly exploring model performance to assess the intrinsic information encoded within the features and given their representativeness, the data itself. Some of these data types may be more amenable to MLM analysis than others. For example, transcriptomics studies are generally higher throughput and produce more available data points for analysis.

Interestingly, MLMs and related techniques have been employed to identify estrous cycle phase based from vaginal cytology (Babaev et al., 2025; Strauss et al., 2024; Q. Wang et al. 2025a, b; Wolcott et al. 2022), however these methods require additional sample collection and it would be useful to be able to identify estrous cycle phase based only from neuron electrophysiology, especially from additional brain regions. An important area of future research will be whether this model or a similar construct could be retrospectively applied to electrophysiological datasets in which the estrous cycle phase was not identified. This data mining approach, if successful, would substantially increase the value of existing datasets. A challenge with this retrospective approach is that the majority of neuroscience research studies do not address sex as an experimental variable (Beery & Zucker, 2011; Lee et al., 2025; Mamlouk et al., 2020; Shansky, 2024; Will et al., 2017; Zucker et al., 2022), much less the estrous cycle (Rocks et al., 2022). Thus, computational techniques for identifying the influence of the estrous cycle upon neuron physiology could exert a high impact if animal sex was documented. We note that the parent study only recorded MSNs from animals with clearly defined phase identifications, so samples from animals that could not be definitively assigned to a phase may be challenging for the MLM and this question could be addressed in future studies. This challenge could be addressed by only assigning neurons to an estrous phase in cases of suitably high confidence, and having a category of “transitional” or “undefined” neuron status in retrospective data mining applications.

While this work focuses on the NAc, neurons from multiple brain regions have demonstrated sensitivity to the estrous cycle, including the hippocampus, amygdala, hypothalamus and others (Blume et al., 2017; Power & Iremonger, 2021; Rocks & Kundakovic, 2023; Wolcott et al., 2025; Woolley & McEwen, 1992). From data generated by our own laboratory, MSNs located in rat caudate-putamen show differences in electrophysiological properties across the estrous cycle, albeit a different pattern than those in the NAc (Willett et al., 2020). A future analysis of the caudate-putamen dataset using MLMs could provide more insights into how the estrous cycle modulates the different aspects of neuronal biophysical processing, and how changes in MSNs in one striatal brain region would compare to those in a different region.

## Conclusion

This study explored the use of MLMs for understanding the influence of the estrous cycle upon the function of NAc MSNs. Across the seven MLMs and three sets of distinct neuronal electrophysiological features, insights based on model success across different feature sets revealed in what capacity the estrous cycle influenced a neuron’s function. It was determined that features extracted from passive membrane recordings resulted in the best model performance, followed by AP, then lastly, mEPSC. Further analysis revealed that input resistance, burst length, and baseline were the most informative features for the best performing model across their respective feature sets, providing more specific insight into what aspects of neuronal processing and communication are modulated by hormones. More broadly, this study represents the use of MLMs as a tool to explore data, leveraging their inherent predictive capacity accessible through performance metrics and feature importances in this case, helping shift towards model interpretability as a tool to extract biological meaning from data without requiring hypothesis-driven statistical frameworks. This use of MLMs may help inform future hypothesis about the underlying mechanisms of processes in a data-driven way, allowing for the elucidation of the impact of different and subtle neuromodulatory variables that interplay to induce a finalized electrophysiological phenotype in a specific neuron type, allowing for additional insight into neuronal variability.

## Supplementary Information

Below is the link to the electronic supplementary material.

Supplementary Material 1

## Data Availability

The parent electrophysiological dataset used in this study is available at the Dryad Data Repository (https://datadryad.org/dataset/doi:10.5061/dryad.k0p2ngfgm). The code generated during this study to analyze the parent dataset is available in the GitHub repository (https://github.com/Armaan-Raina/Estrous-Phase-Classification).
