# Supplementary material for: Application of Machine Learning Models to Identify Differences in Neural Electrophysiological Properties Across Estrous Cycle Phases
Source: Neuroinformatics. 2026 Sep 1;24(3):57. doi: 10.1007/s12021-026-09814-0 (PMC13534189; doi:10.1007/s12021-026-09814-0)
Supplement: Supplementary file 1 [file 12021_2026_9814_MOESM1_ESM.docx]

**Supplemental tables**

Table S1.
.

| Panel | Model | Comparison | Difference (pp) | 95% CI | p-adj | Significance |
| --- | --- | --- | --- | --- | --- | --- |
| A | RFC | Passive - Action Potentials | 11.33 | [9.55, 13.11] | <0.0001 | ** |
| A | RFC | mEPSC - Action Potentials | -12.72 | [-14.50, -10.94] | <0.0001 | ** |
| A | RFC | mEPSC - Passive | -24.06 | [-25.84, -22.28] | <0.0001 | ** |
| A | GBC | Passive - Action Potentials | 17.24 | [15.46, 19.02] | <0.0001 | ** |
| A | GBC | mEPSC - Action Potentials | -6.87 | [-8.65, -5.09] | <0.0001 | ** |
| A | GBC | mEPSC - Passive | -24.11 | [-25.89, -22.33] | <0.0001 | ** |
| A | LR | Passive - Action Potentials | 0.75 | [-1.03, 2.53] | 0.9919 | n.s. |
| A | LR | mEPSC - Action Potentials | -14.62 | [-16.40, -12.84] | <0.0001 | ** |
| A | LR | mEPSC - Passive | -15.38 | [-17.16, -13.60] | <0.0001 | ** |
| A | KNN | Passive - Action Potentials | 2.54 | [0.76, 4.32] | 0.00020 | * |
| A | KNN | mEPSC - Action Potentials | -25.61 | [-27.39, -23.83] | <0.0001 | ** |
| A | KNN | mEPSC - Passive | -28.15 | [-29.93, -26.37] | <0.0001 | ** |
| A | MLP | Passive - Action Potentials | 7.32 | [5.54, 9.10] | <0.0001 | ** |
| A | MLP | mEPSC - Action Potentials | -14.32 | [-16.10, -12.54] | <0.0001 | ** |
| A | MLP | mEPSC - Passive | -21.64 | [-23.42, -19.86] | <0.0001 | ** |
| A | SVC | Passive - Action Potentials | 0.49 | [-1.29, 2.27] | 1.0000 | n.s. |
| A | SVC | mEPSC - Action Potentials | -15.27 | [-17.04, -13.49] | <0.0001 | ** |
| A | SVC | mEPSC - Passive | -15.76 | [-17.54, -13.98] | <0.0001 | ** |
| A | DTC | Passive - Action Potentials | 15.0 | [13.22, 16.78] | <0.0001 | ** |
| A | DTC | mEPSC - Action Potentials | -16.23 | [-18.01, -14.45] | <0.0001 | ** |
| A | DTC | mEPSC - Passive | -31.23 | [-33.01, -29.45] | <0.0001 | ** |
| B | Action Potentials | GBC - RFC | -6.16 | [-7.94, -4.38] | <0.0001 | ** |
| B | Action Potentials | LR - RFC | -14.7 | [-16.48, -12.92] | <0.0001 | ** |
| B | Action Potentials | KNN - RFC | 0.88 | [-0.90, 2.66] | 0.9571 | n.s. |
| B | Action Potentials | MLP - RFC | -0.92 | [-2.70, 0.86] | 0.9363 | n.s. |
| B | Action Potentials | SVC - RFC | -7.75 | [-9.53, -5.97] | <0.0001 | ** |
| B | Action Potentials | DTC - RFC | -5.02 | [-6.80, -3.25] | <0.0001 | ** |
| B | Action Potentials | LR - GBC | -8.54 | [-10.32, -6.76] | <0.0001 | ** |
| B | Action Potentials | KNN - GBC | 7.05 | [5.27, 8.83] | <0.0001 | ** |
| B | Action Potentials | MLP - GBC | 5.24 | [3.46, 7.02] | <0.0001 | ** |
| B | Action Potentials | SVC - GBC | -1.59 | [-3.37, 0.19] | 0.1419 | n.s. |
| B | Action Potentials | DTC - GBC | 1.14 | [-0.64, 2.92] | 0.7111 | n.s. |
| B | Action Potentials | KNN - LR | 15.58 | [13.80, 17.36] | <0.0001 | ** |
| B | Action Potentials | MLP - LR | 13.78 | [12.00, 15.56] | <0.0001 | ** |
| B | Action Potentials | SVC - LR | 6.95 | [5.17, 8.73] | <0.0001 | ** |
| B | Action Potentials | DTC - LR | 9.68 | [7.90, 11.46] | <0.0001 | ** |
| B | Action Potentials | MLP - KNN | -1.81 | [-3.59, -0.03] | 0.0427 | * |
| B | Action Potentials | SVC - KNN | -8.64 | [-10.42, -6.86] | <0.0001 | ** |
| B | Action Potentials | DTC - KNN | -5.91 | [-7.69, -4.13] | <0.0001 | ** |
| B | Action Potentials | SVC - MLP | -6.83 | [-8.61, -5.05] | <0.0001 | ** |
| B | Action Potentials | DTC - MLP | -4.1 | [-5.88, -2.32] | <0.0001 | ** |
| B | Action Potentials | DTC - SVC | 2.73 | [0.95, 4.51] | <0.0001 | ** |
| C | Passive | GBC - RFC | -0.25 | [-2.03, 1.53] | 1.0000 | n.s. |
| C | Passive | LR - RFC | -25.28 | [-27.06, -23.50] | <0.0001 | ** |
| C | Passive | KNN - RFC | -7.91 | [-9.69, -6.13] | <0.0001 | ** |
| C | Passive | MLP - RFC | -4.94 | [-6.72, -3.16] | <0.0001 | ** |
| C | Passive | SVC - RFC | -18.59 | [-20.37, -16.81] | <0.0001 | ** |
| C | Passive | DTC - RFC | -1.36 | [-3.14, 0.42] | 0.3813 | n.s. |
| C | Passive | LR - GBC | -25.03 | [-26.81, -23.25] | <0.0001 | ** |
| C | Passive | KNN - GBC | -7.66 | [-9.44, -5.88] | <0.0001 | ** |
| C | Passive | MLP - GBC | -4.68 | [-6.46, -2.90] | <0.0001 | ** |
| C | Passive | SVC - GBC | -18.34 | [-20.12, -16.56] | <0.0001 | ** |
| C | Passive | DTC - GBC | -1.11 | [-2.89, 0.67] | 0.7544 | n.s. |
| C | Passive | KNN - LR | 17.37 | [15.59, 19.15] | <0.0001 | ** |
| C | Passive | MLP - LR | 20.34 | [18.56, 22.12] | <0.0001 | ** |
| C | Passive | SVC - LR | 6.69 | [4.91, 8.47] | <0.0001 | ** |
| C | Passive | DTC - LR | 23.92 | [22.14, 25.70] | <0.0001 | ** |
| C | Passive | MLP - KNN | 2.98 | [1.20, 4.76] | <0.0001 | ** |
| C | Passive | SVC - KNN | -10.68 | [-12.46, -8.90] | <0.0001 | ** |
| C | Passive | DTC - KNN | 6.55 | [4.77, 8.33] | <0.0001 | ** |
| C | Passive | SVC - MLP | -13.66 | [-15.44, -11.88] | <0.0001 | ** |
| C | Passive | DTC - MLP | 3.58 | [1.80, 5.35] | <0.0001 | ** |
| C | Passive | DTC - SVC | 17.23 | [15.45, 19.01] | <0.0001 | ** |
| D | mEPSC | GBC - RFC | -0.31 | [-2.09, 1.47] | 1.0000 | n.s. |
| D | mEPSC | LR - RFC | -16.6 | [-18.38, -14.82] | <0.0001 | ** |
| D | mEPSC | KNN - RFC | -12.01 | [-13.78, -10.23] | <0.0001 | ** |
| D | mEPSC | MLP - RFC | -2.52 | [-4.30, -0.74] | 0.00023 | * |
| D | mEPSC | SVC - RFC | -10.3 | [-12.07, -8.52] | <0.0001 | ** |
| D | mEPSC | DTC - RFC | -8.53 | [-10.31, -6.75] | <0.0001 | ** |
| D | mEPSC | LR - GBC | -16.29 | [-18.07, -14.51] | <0.0001 | ** |
| D | mEPSC | KNN - GBC | -11.7 | [-13.48, -9.92] | <0.0001 | ** |
| D | mEPSC | MLP - GBC | -2.21 | [-3.99, -0.43] | 0.0026 | * |
| D | mEPSC | SVC - GBC | -9.99 | [-11.77, -8.21] | <0.0001 | ** |
| D | mEPSC | DTC - GBC | -8.22 | [-10.00, -6.44] | <0.0001 | ** |
| D | mEPSC | KNN - LR | 4.6 | [2.82, 6.38] | <0.0001 | ** |
| D | mEPSC | MLP - LR | 14.08 | [12.30, 15.86] | <0.0001 | ** |
| D | mEPSC | SVC - LR | 6.31 | [4.53, 8.09] | <0.0001 | ** |
| D | mEPSC | DTC - LR | 8.07 | [6.29, 9.85] | <0.0001 | ** |
| D | mEPSC | MLP - KNN | 9.48 | [7.70, 11.26] | <0.0001 | ** |
| D | mEPSC | SVC - KNN | 1.71 | [-0.07, 3.49] | 0.0749 | n.s. |
| D | mEPSC | DTC - KNN | 3.47 | [1.69, 5.25] | <0.0001 | ** |
| D | mEPSC | SVC - MLP | -7.77 | [-9.55, -5.99] | <0.0001 | ** |
| D | mEPSC | DTC - MLP | -6.01 | [-7.79, -4.23] | <0.0001 | ** |
| D | mEPSC | DTC - SVC | 1.76 | [-0.02, 3.54] | 0.0550 | n.s. |
